# To catch a quake

**DOI:** 10.1038/s41467-018-04790-9

**Published:** 2018-07-03

**Authors:** Elizabeth S. Cochran

**Affiliations:** Earthquake Science Center, U.S. Geological Survey, Pasadena, CA 91106 USA

## Abstract

A revolution in seismic detection technology is underway, capturing unprecedented observations of earthquakes and their impacts. These sensor innovations provide real-time ground shaking observations that could improve emergency response following damaging earthquakes and may advance our understanding of the physics of earthquake ruptures.

## Introduction

On average, at least one earthquake of magnitude 7 or greater occurs each month somewhere around the globe. Many of these earthquakes go largely unnoticed as they occur in remote locations, but when a major earthquake happens near an urban centre it can result in substantial loss of life and economic losses. For example, the 2011 M9.0 Tohoku earthquake resulted in more than $300 billion USD in losses due to the earthquake and tsunami^1^, and the 2010 M7.0 Haiti earthquake caused significant loss of life with over 100,000 deaths^1^. The impacts of earthquakes are typically greater in developing countries where large populations centres are located in regions of high seismic hazard and the traditional building stock is more vulnerable to earthquake shaking^1^. Understanding the physics of how, when, and why large, potentially damaging earthquakes occur is critical for mitigating their effects. Yet, there are essentially no direct measurements of fault slip at the depths at which earthquakes start, typically a few to tens of kilometres below the Earth’s surface. Seismometers can accurately detect the displacement, velocity, or acceleration of the ground surface due to permanent shifts resulting from fault movement or the transient oscillations as seismic waves pass under a site. However, these data typically provide only scant observations as instruments are spaced too widely or data are not continuous in time.

Cost also presents a large impediment to long-term, dense (stations spaced at less than 10–20 km) ground motion observations. Research-grade, high-resolution seismic sensors are a fairly niche market with limited customers. These expensive specialty instruments are placed in long-term installations that are hardened to withstand strong shaking from earthquakes. Just one of these stations costs tens of thousands of dollars to build and equip, including sensors, on-site data acquisition systems, telecommunications, and back-up power. Multiple ground shaking measurements are required to locate and characterise an earthquake; and, in the event of a major earthquake the data are critical for determining the severity of shaking across the impacted region in order to guide emergency response to the hardest hit areas. Furthermore, earthquake early warning systems that rapidly detect earthquakes could provide critical warnings to affected populations prior to the arrival of damaging seismic waves, but these systems require dense instrumentation as every additional station improves the speed and accuracy of the alerts. Even the densest seismic networks in the world typically do not have more than one sensor every ~20 km, which can cost hundreds of thousands of dollars or more each year to operate and maintain. Operating a state-of-the-art seismic network can be beyond the reach of many developing countries leaving them especially vulnerable given the lack of data to understand local seismic hazards.

However, today earthquake sensing is in the midst of a renaissance as the methods available to more densely observe earthquake ground motions have expanded by leveraging both new types of purpose-built seismic instrumentation and new sensors in the built environment.

## The revolution in seismic instrumentation

**Fig. 1:**
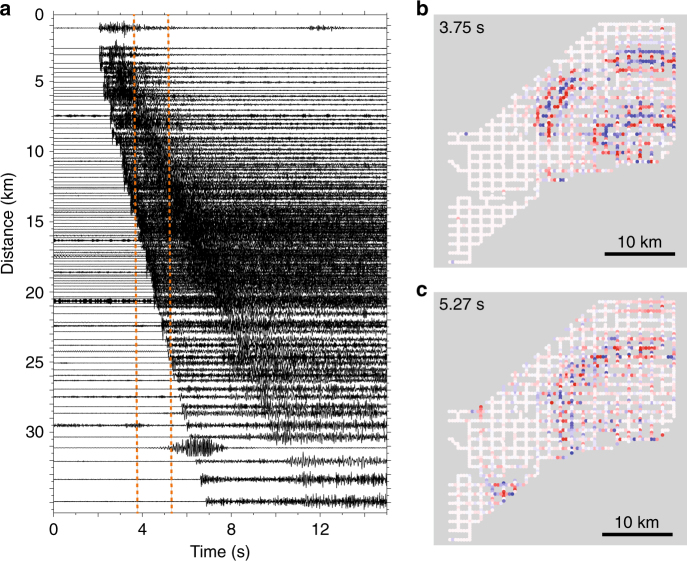
Example of a M3.0 earthquake recorded by an 1800 sensor nodal array in Oklahoma. **a** Waveforms of the earthquake along one line of sensors; the times of the snapshots shown in **b** and **c** are shown by orange dashed lines, **b** Wavefield observed on 1800 nodes at 3.75 seconds after the earthquake origin time; red and blue colors denote when ground is moving up or down, respectively, and **c** Wavefield at 5.27 seconds after the origin time. (Image Credit: S. L. Dougherty, USGS)

Advances in sensor and communication technologies are opening up opportunities for very dense purpose-built seismic sensors with lower per-station costs. The energy industry uses seismic sensors to undertake surveys of shallow Earth structure and through recent innovations cabled sensor systems have been converted into wireless nodal instruments^2^. These sensors eliminate the need for transport and installation of the heavy cables that once connected sensors together, and have batteries that last 30 or more days, making these systems attractive for use in earthquake studies. Without cables, the individual sensors are lightweight and easy to transport to remote locations so that station layouts can be better tailored to specific study targets. A 6-month deployment of 5400 sensors over a ~100 km^2^ area in Long Beach, California was deployed by industry, but data were made available to academic researchers. The data showed unexpected slip occurring in the deep roots of faults^3^ and provided insights into how shaking may vary across the region during a large earthquake by imaging the shallow subsurface^4^. A recent experiment on Mount St. Helens deployed a spiderweb of 904 nodes for 2 weeks providing an order of magnitude increase in the number of earthquakes observed beneath the active volcano compared to the existing traditional seismic network^5^. And, nodal arrays in Oklahoma are being used to track small earthquakes moving along previously undetected fault structures that are induced by injection of wastewater into deep wells in areas of oil and gas production^6,7^ (Fig. 1).

While nodal array systems provide extremely dense sensor arrays at significantly lower costs than previously possible, they can only record ground motions over relatively short time periods (weeks to months). The advent of low-cost, ‘personal’ seismometers provides opportunities for long-term installations at only a few hundred dollars each, a fraction of the cost of a typical seismic station^8,9^. These systems utilise compact computers, often called plug computers, that are low power and inexpensive and take advantages of new advances in commercial-grade accelerometer sensors. The accelerometer chips are inexpensive as they are produced in bulk for use in activating car airbags, rotating smart phone screens, and gaming systems. While the sensors are lower resolution than scientific-grade instruments, they are adequate for recording moderate to strong ground motions and cheap enough to allow for building dense networks to observe ground motions on a block-by-block scale. Sensors installed by Community Seismic Network inside buildings have measured how buildings sway in response to passing earthquake waves providing real-world data to test the models that structural engineers have typically relied upon^8^. Raspberry Shake sensors are adding to local observations of ground motions from moderate to large earthquakes, including in countries with more limited seismic monitoring infrastructure^9^.

## Everyday sensors

**Fig. 2:**
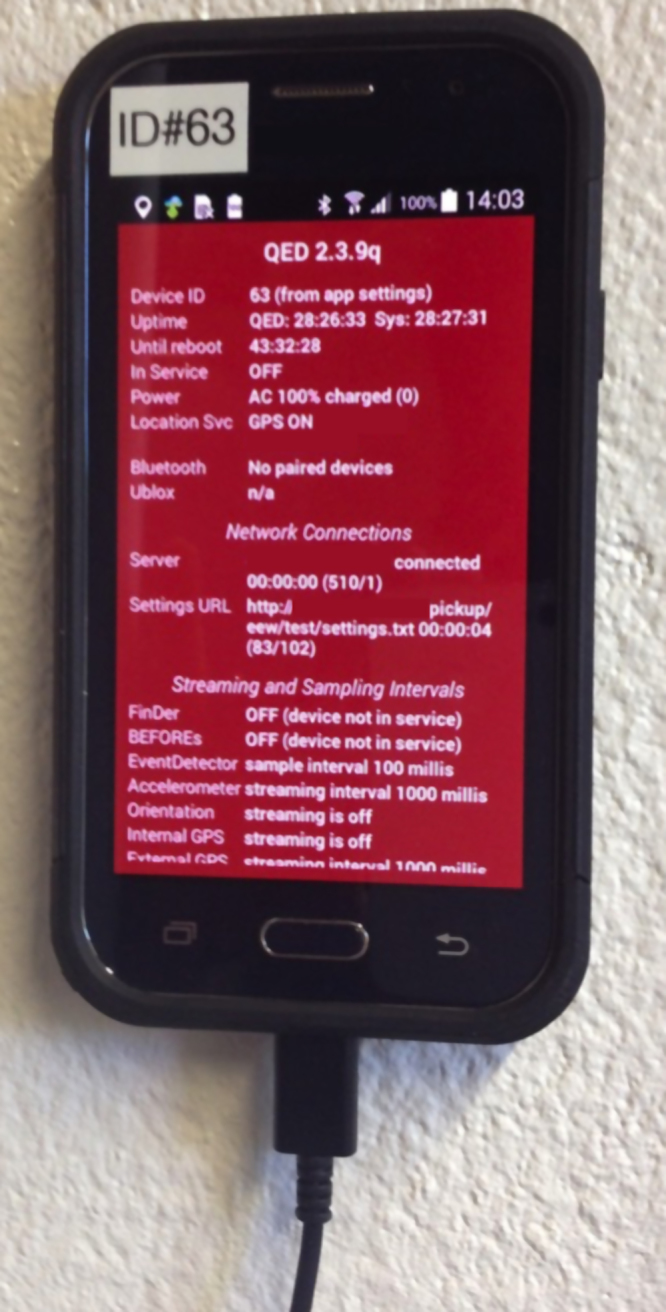
Smartphone set up as a dedicated seismic sensor to record earthquakes in Chile. (Image Credit: B. A. Brooks, USGS)

With plunging sensor prices, accelerometers are being integrated into many everyday objects making the idea of harnessing interconnected devices, or the so-called Internet of Things (IoT), for earthquake detection ever more attainable. Several groups have been working on collecting and combining data from consumer devices. The Quake Catcher Network pioneered the concept, by exploiting commercial-grade accelerometer inside laptops and smartphones and using distributed computing techniques to record data and detect quakes^10^. Rapid deployment of these sensors following the 2010 M8.8 Maule, Chile and the 2010 M7.2 Darfield, New Zealand earthquakes expanded the number of available observations from a few tens of research-grade sensors to hundreds of (lower-resolution) sensors used for rapid event detection, characterisation of local geology, and details about the earthquake sources^11–13^. MyShake also uses smartphones but integrates machine-learning techniques to separate quakes from other movement (walking, etc.)^14^. And, a system deployed in Chile (Fig. 2) uses the smartphone’s accelerometer chip to capture ground accelerations and a Global Positioning System (GPS) chip to capture ground displacements^15^, with application for earthquake and tsunami warning^16^.

As described by Jousset et al.^17^ in a recent publication, fibre optic cables used for communication can be transformed into a series of sensors through the use of laser pulses that are backscattered in the fibre and recorded by a receiver. These sensors measure strain, i.e., very small changes in the length of the fibre cable (and the ground in which the fibre is installed) from extension and compression as seismic waves pass. Effective sensor spacing on the order of 1 to 10 m can be achieved, providing ten to fifty times more sensors over a given distance than even nodal systems. A 15-km-long fibre-optic cable with 4-m sensor spacing is providing new images of extraordinary resolution that show the deformation of faults in Iceland and seismic waves trapped within fault zones (Jousset et al.^17^). Fibre optic cables have also successfully recorded small earthquakes in Alaska and California at resolutions similar to scientific-grade equipment^17,18^. However, although promising, this approach requires significant development in order to translate and calibrate the data for widespread use.

## The future of seismic observations

Each of these novel ground motion sensor networks provide incredible opportunities for recording rich datasets with which to study the movement of waves through the Earth and to decipher the inner workings of faults. Low-cost sensors may transform earthquake science in countries at significant risk of damaging earthquakes, but which may not be able to afford the high cost of installing and maintaining traditional seismic networks. There are still limitations to the resolution of the sensors, interpretation of the resulting data, and efficient management of the resulting large data volumes. Despite the limitations, I look forward to a time when smoke detectors and smart gas meters, or even perhaps millions of toasters provide records useful for seismology.
